# Exploring of the property of epoxy resins based on diselenide and disulfide dynamic linkers

**DOI:** 10.3389/fchem.2022.991010

**Published:** 2022-09-05

**Authors:** Xiao Wei, Feng Liu, Xinru Guo, Fei Gao, Yingjia Li, Dongtao Zhu, Zhi Zhou, Liang Shen

**Affiliations:** Jiangxi Engineering Laboratory of Waterborne Coating, School of Chemistry and Chemical Engineering, Jiangxi Science and Technology Normal University, Nanchang, China

**Keywords:** epoxy resins, aromatic diselenide, aromatic disulfide, self-healing property, dynamic covalent bonds

## Abstract

Over the last decade, there has been a lot of interest in incorporating dynamic covalent bonds (DCBs) into epoxy resins. Because diselenide and disulfide bonds have similar properties, they are frequently used as DCBs in self-healing epoxy networks. In this paper, we present diselenide and disulfide dynamic linkers containing epoxy networks by analyzing the effects of mechanical properties, thermal stability, activation energies, and self-healing properties. The glass transition temperature (*T*_g_) values, mechanical properties, crosslinking density (*v*_*e*_), and thermal stability of disulfide linkers networks were higher than those of diselenide linkers networks, according to our research. The activation energies of disulfide linkers were higher than those of diselenide linkers (up to 14 kJ/mol), but their healing efficiency was lower than that of the diselenide network. These findings demonstrate the advantages of diselenide and disulfide dynamic linkers in epoxy networks systems, as well as a method for designing and preparing the appropriate diselenide dynamic linkers or disulfide dynamic linkers incorporated into epoxy networks for the appropriate application and processing technology.

## Introduction

Because of their excellent chemical resistance, thermal stability, and mechanical properties, epoxy resins are widely used in coatings, adhesives, electronics industry machinery, and other fields (Chung 2019; Luo et al., 2022). These benefits, however, limit the service life of materials (Le Goff et al., 2020). In recent years, the introduction of dynamic covalent bonds (DCBs) into polymers has got a lot of attention because it can extend their lifetime and improve their reliability (Burattini et al., 2010; Billiet et al., 2013; Wang et al., 2019). Transesterification (Kar et al., 2020), Diels–Alder reaction (Chang et al., 2021), radical reshuffling (Imato et al., 2012), olen metathesis (He et al., 2019a), siloxane equilibration (Tretbar et al., 2019), imine (García Jeannette et al., 2014) or hydrazine formation (Liu et al., 2012), aliphatic disulfide exchange (Lafont et al., 2012) and thiol–nanoparticle exchange (Martín et al., 2012), and other methods have been developed to produce DCBs. These DCBs open up possibilities for the use of self-healing materials.

Both diselenide bonds and disulfide bonds are DCBs, and they are frequently used as self-healing, dynamic covalent bonds (Ji et al., 2015; Kim et al., 2018; Li et al., 2018; Ling et al., 2018). Much research has been conducted on the use of disulfide bonds and diselenide bonds to prepare self-healing epoxy resins, with promising results (Azcune and Odriozola 2016). Odriozola et al. introduce 2-aminophenyl disulfide into epoxy resin, which exhibits good mechanical properties as well as processability, reparability, and recyclability (de Luzuriaga et al., 2016). Zeng et al. demonstrated a novel epoxy material derived from the reaction of 4,4′-dithiodiphenylamine with epoxidized soybean oil, and the mechanical properties were almost recovered completely when two broken parts of materials were welded together (Liu et al., 2020). Liang et al. introduced dynamic diselenide bonds into epoxy thermosets, which have good self-healing and shape-memory (Liu et al., 2022). Although dynamic disulfide bonds and dynamic diselenide bonds can be used as DCBs of epoxy systems, the effects of crosslinkers on the mechanical properties, thermal stability, and self-healing of epoxy materials have not been studied.

The effect of dynamic disulfide bonds and dynamic diselenide bonds containing epoxy networks is investigated in this study. The glass transition temperature (*T*_g_) values, mechanical properties, crosslinking density (*v*_*e*_), thermal stability, and activation energies of disulfide linkers networks were higher than those of diselenide linkers networks, but the healing efficiency was lower than diselenide linkers networks.

## Experimental

### Materials

Bisphenol A epoxy resins (128), 2-aminophenyl disulfide (2-DS), 4-Aminophenyl disulfide (4-DS), 2-Iodoaniline and 4-Iodoanline were purchased from Energy Chemical (Shanghai, China). CuO nanopowders, KOH and selenium powder were provided by Aladdin Bio-Chem Technology. Poly (ethylene glycol) diglycidyl ether (DER736) was obtained by Macklin Biochemical. Petroleum ether, dimethyl sulfoxide (DMSO), ethyl acetate and dichloromethane were supplied by Guangzhou reagent factory. All the materials were used as received.

### Synthesis of diselenide linkers (4-DSe and 2-DSe)


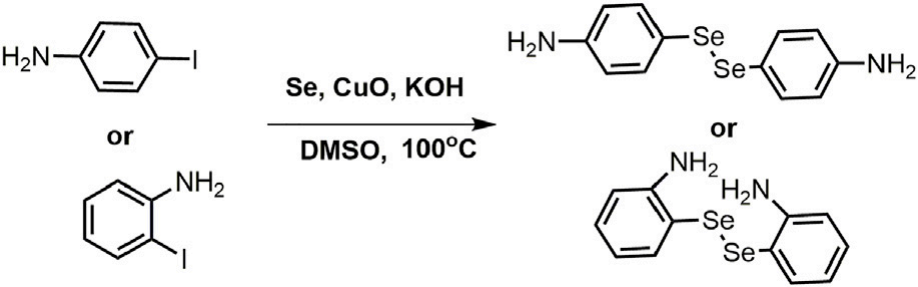


A solution of 1 equiv p-Iodoaniline and 5 equiv selenium powder in dry DMSO (100 ml) was stired for 30 min at room temperature. The mixture solution was then supplemented with 2 equiv KOH and 10% *wt* CuO nanoparticles and stirred for another 30 min. Finally, the above reactions were transferred to an oil bath at 100°C in a N_2_ atmosphere for 24 h. The reaction was monitored by TLC. Following the completion of the reaction, the crude products were extracted by vacuum, and the pure diselenides linkers were purified by column chromatography separation (petroleum ether/ethyl acetate = 1:1, v/v) with the yield of 52% (4-DSe linker) and 47% (2-DSe linker). 4-DSe: ^1^H NMR (400 MHz, CDCl_3_): δ(ppm) = 7.38 (d,j, 4H), 6.57 (d,j, 4H), 3.50 (s, 4H); ^13^C NMR (CDCl_3_, 100 MHz): δ(ppm) = 146.93, 136.06, 134.51, 115.45; 2-DSe: ^1^H NMR (400 MHz, CDCl_3_): δ(ppm) = 7.35 (d,d, 2H), 7.16–7.12 (m, 2H), 6.73 (d, d, 2H), 6.58–6.54 (m, 2H), 2.95 (s, 4H); ^13^C NMR (CDCl_3_, 100 MHz): δ(ppm) = 148.12, 137.75,130.97, 117.77, 114.10.

### Synthesis of dynamic epoxy resins networks

**SCHEME 1:**
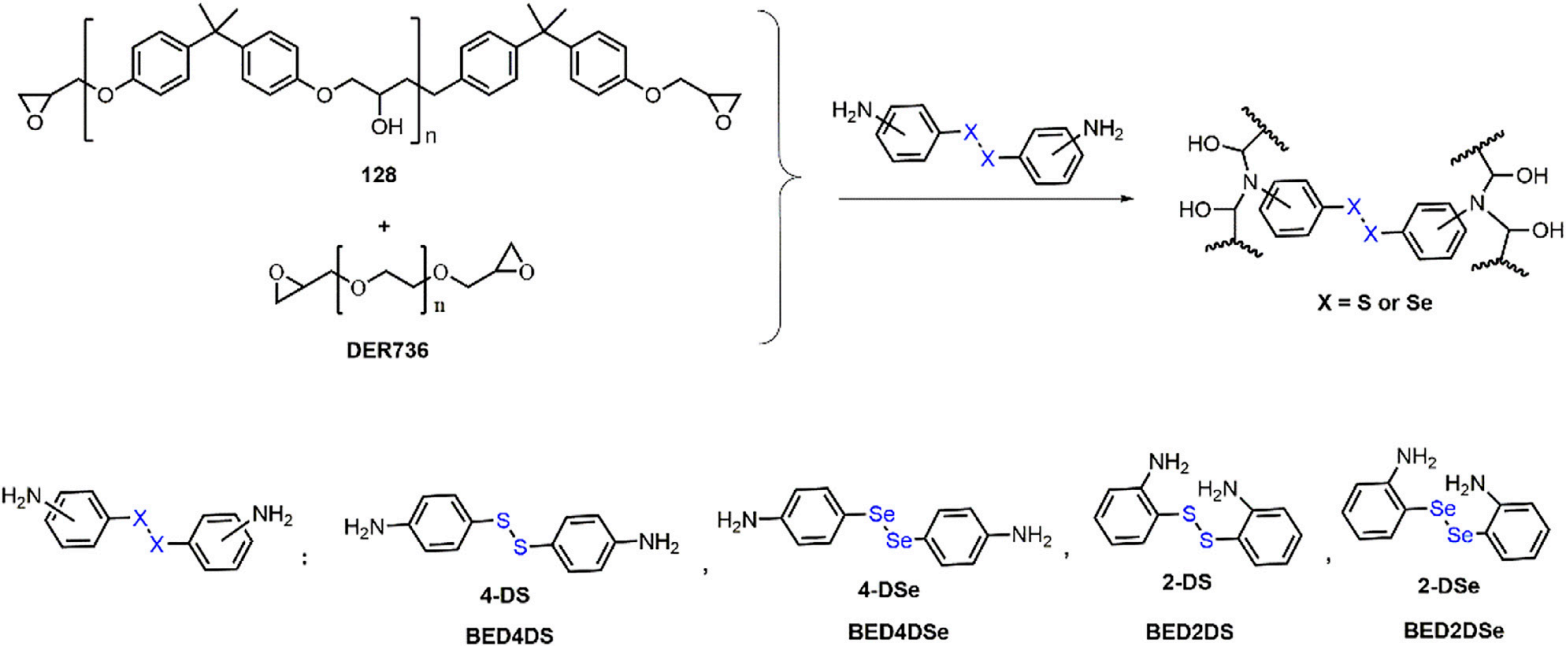
The synthetic route of the dynamic epoxy resin networks.

Scheme 1 and Table 1 depict the synthetic route and formation of dynamic epoxy resin networks respectively. In the glass bottle, Bisphenol A epoxy resins 128 (15 mmol) and DER736 (22.5 mmol) were mixed. The crosslinker 20.8 mmol (4-DS, 4-DSe, 2-DS, or 2-DSe) solution in 3 ml DMF was then added. The resulting mixture was placed on a PTFE mold (80 mm* 80 mm* 15 mm), and the curing process was monitored using FTIR spectroscopy for 24 h at 150°C.

**TABLE 1:** The formulation of the dynamic epoxy resin networks.

Resin	128 (mmol)	DER736 (mmol)	crosslinker (mmol)	Amine/Epoxy
BED4DS	15	22.5	20.8 (4-DS)	1/0.9
BED4DSe	15	22.5	20.8 (4-DSe)	
BED2DS	15	22.5	20.8 (2-DS)	
BED2DSe	15	22.5	20.8 (2-DSe)	

### Characterization

NMR measurements were carried out at 400 MHz on a Bruker AV-400 NMR instrument with CDCl_3_ as the solvent. A Bruker-Veretex70 spectrometer was used to obtain the FTIR spectra. The differential scanning calorimeter (DSC) measurements were carried out on TA-Q200 (United States) from −20°C to 110°C at a heat rate of 10°C/min. Tensile tests were carried out using a dynamic mechanical thermal analysis (DMTA, TA-Q800, United States) under 50 mm min^−1^ of the cross-head rate with a range of −10°C to 130°C at a heating rate of 5°C min^−1^. Thermogravimetric analysis (TGA-Q50, United States) was performed under nitrogen from 25°C to 700°C at a heat rate of 10°C/min. The dynamic mechanical analysis (DMA) machine was also used for stress-relaxation experiments and activity energy experiments (TA-Q800, United States). The stress-relaxation curves of the film were tested at different temperatures (90°C, 100°C, 110°C, and 120°C) with a constant strain of 2% (at appropriate size). The gel fraction (W) was calculated using the following formula: W = M_2_/M_1_ × 100%. M_1_ represents the weight of the original sample, while M_2_ represents the weight of the dried samples (the constant weight obtained by drying the epoxy resinsat 80°C for 24 h after immersing them in acetone for 24 h. The self-healing photographs were recorded by using metallurgical microscopy (CMY-310).

## Results and discussion

### Characterization of dynamic epoxy resin networks

**FIGURE 1:**
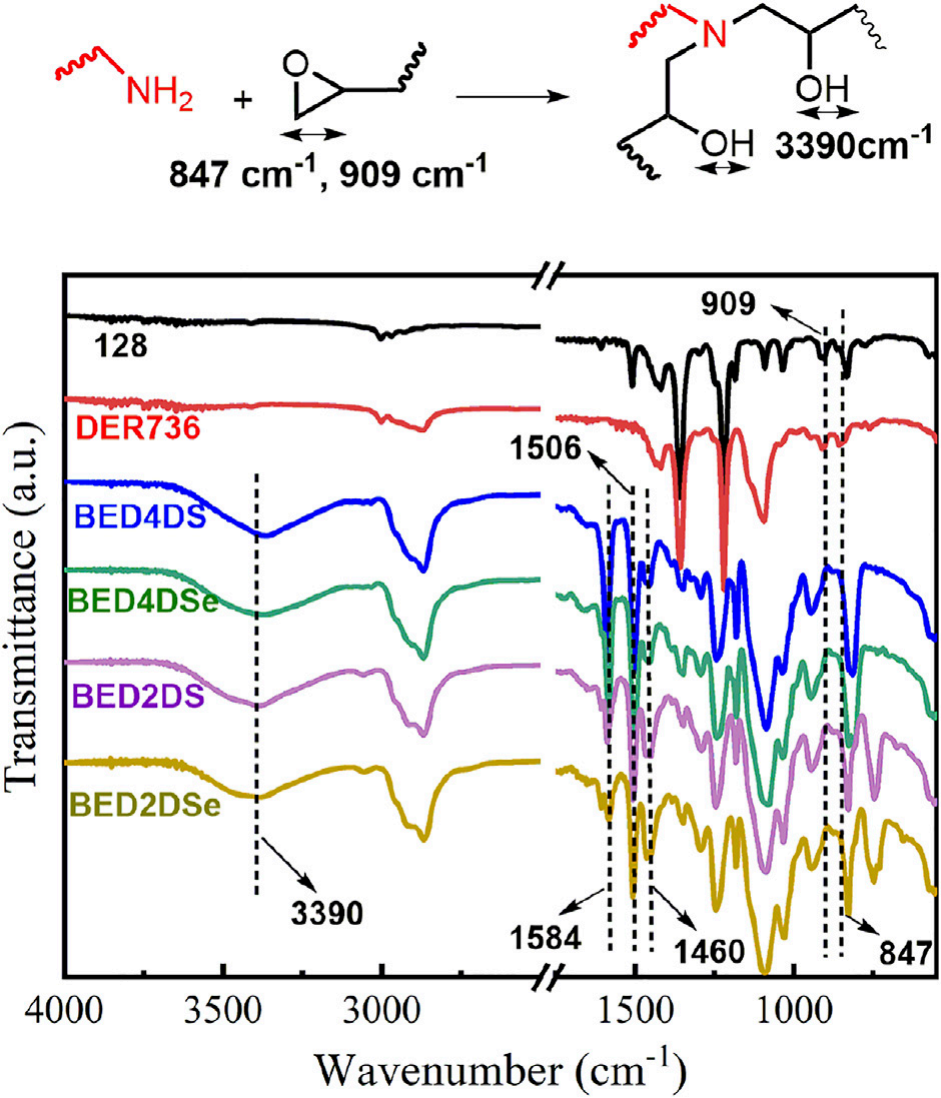
FTIR spectra of the dynamic epoxy resin networks.

FTIR spectra confirmed the chemical structure of dynamic epoxy resin networks (BED4DS, BED4DSe, BED2DS, and BED2DSe). As shown in Figure 1, the epoxy group stretching peaks at 847 cm^−1^ and 909 cm^−1^ (128 and DER736) vanished, while benzene ring absorption peaks at 1,584, 1,506 and 1,460 cm^−1^ and the hydroxyl groups at 3,390 cm^−1^ appeared in dynamic epoxy resin networks. These findings were consistent with previous findings (Rosu et al., 2015; Le Goff et al., 2020; Ruiz de Luzuriaga et al., 2022). Furthermore, because gel content is an important network index (Luo et al., 2020), the gel contents of dynamic epoxy resin networks are high (higher than 96%). These findings demonstrated the formation of dynamic epoxy resin networks.

**FIGURE 2:**
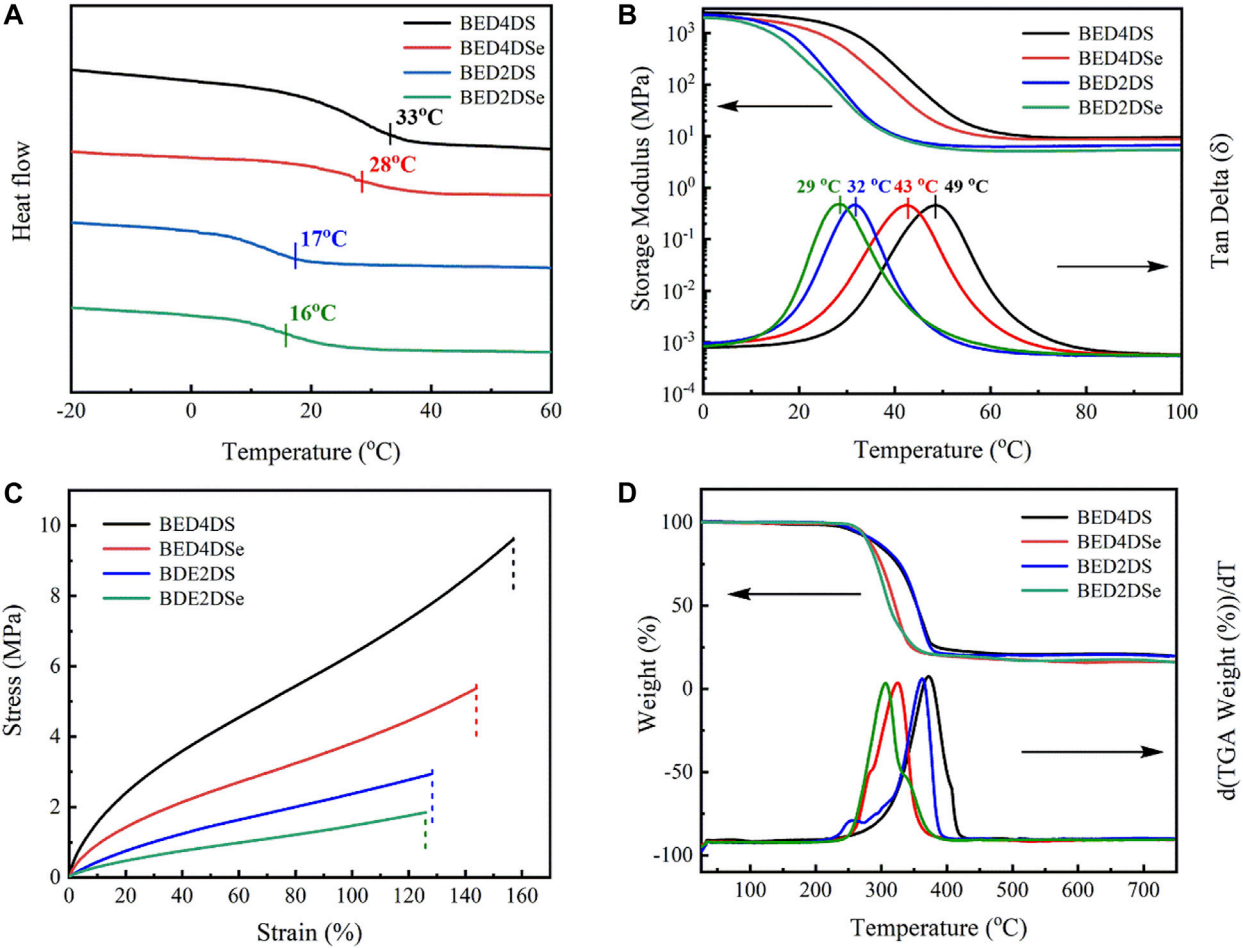
The thermal and mechanical properties of dynamic epoxy resin networks **(A)** DSC curves; **(B)** stress-strain curves; **(C)** DMA curves; **(D)** TGA curves of dynamic epoxy resin networks.

The glass transition temperature (*T*_g_) was determined using DSC. *T*_g_ values for BED4DS, BED4DSe, BED2DS, and BED2DSe, as shown in Figure 2A and Table 2, were 33, 28, 17, and 16°C respectively. DSC confirmed that all of the epoxy groups had been completely cured because there was no more curing exothermic peak observed (Zhou et al., 2018). When these four dynamic epoxy resin networks were compared, the *T*_g_ values of p-phenylamino networks (BED4DS and BED4DSe) were higher than o-phenylamino networks (BED2DS and BED2DSe), which may be attributed to the steric hindrance of o-phenylamino linkers. And the *T_g_* values of disulfide linkers networks (BED4DS and BED2DS) were slightly higher than those of diselenide linkers networks (BED4DSe and BED2DSe), which could be attributed to the lower bond energies of diselenide linkers networks (Fan et al., 2018).

**TABLE 2:** The gel contents, and thermal and mechanical properties dates of the dynamic epoxy resin networks.

Sample	Gel content (%)	*T*_*g*_ (DMA) (°C)	*T*_*g*_ (DSC) (°C)	*E′* at *T*_g_ +50 °C (MPa)	Crosslinking (*v*_*e*_) (mol/m^3^)	Young’s modulus (MPa)	Stress at break (MPa)	Elongation at break (%)
BED4DS	97	49	33	9.53	1,186	206.26 ± 873	9.59	157
BED4DSe	95	43	28	8.67	1,100	133.01 ± 5.38	5.25	144
BED2DS	96	32	17	6.52	854	54.47 ± 1.50	2.92	128
BED2DSe	96	29	16	5.25	706	27.62 ± 3.84	1.85	126

Dynamic mechanical thermal analysis (DMTA) was performed on the samples (BED4DS, BED4DSe, BED2DS, and BED2DSe) and the results are shown in Figure 2 and Table 2. Figure 2B shows that as the temperature increased, all of the sample’s storage modulus decreased rapidly and eventually stabilized. Furthermore, we can see that only one peak was observed in each case of the loss factors, indicating that the samples have homogeneous properties, and the peak maximum value can be used as the *T*_g_(Zuo et al. 2019; Chen et al. 2020; Guo et al. 2021). In previous reports, the crosslinking density (*v*_*e*_) was calculated using the equation (Li et al., 2019; Zhu et al., 2020a; Zhu et al., 2020b).

$$\nu_{e}=E\prime /\mathrm{3RT}$$ (1)

E′ denotes the storage modulus at *T*_g_+50°C, R the gas universal constant, and T the *T*_g_ temperature. Table 2 contained a list of all the dates. When these samples are compared, BED4DS has the highest *T*_g_ and *v*_*e*_ *,* while BED2DSe has the lowest *T*_g_ and *v*_*e*_. These results could be attributed to the low steric hindrance and the fact that the selenium atom has a larger radius than the sulfur atom (He et al., 2019b; Qian et al., 2019).

Dynamic mechanical analysis (DMA) was used to characterize the mechanical properties of these samples, as shown in Figure 2C and Table 2. The p-phenylamino networks (BED4DS and BED4DSe) clearly have a higher Young’s modulus and tensile strength, while the o-phenylamino networks (BED2DS and BED2DSe) have a lower Young’s modulus and tensile strength. The disulfide linkers (BED4DS and BED2DS) of samples were slightly higher than the diselenide linkers (BED4DSe and BED2DSe), which could be attributed to the crosslinking density (*v*_*e*_). (Zhang et al., 2019).

Figure 2D and Table 2 show that the thermal stability of the samples. As we can see, all of the samples have reached the thermal degradation stage and are stable up to 250°C. The degradation temperature of BED4DSe and BED2DSe exhibited lower than BED4DS and BED2DS. The results can be attributed to the diselenide bond’s low bond energy (Liu et al., 2022).

### Stress-relaxation behaviors of dynamic epoxy resin networks

**FIGURE 3:**
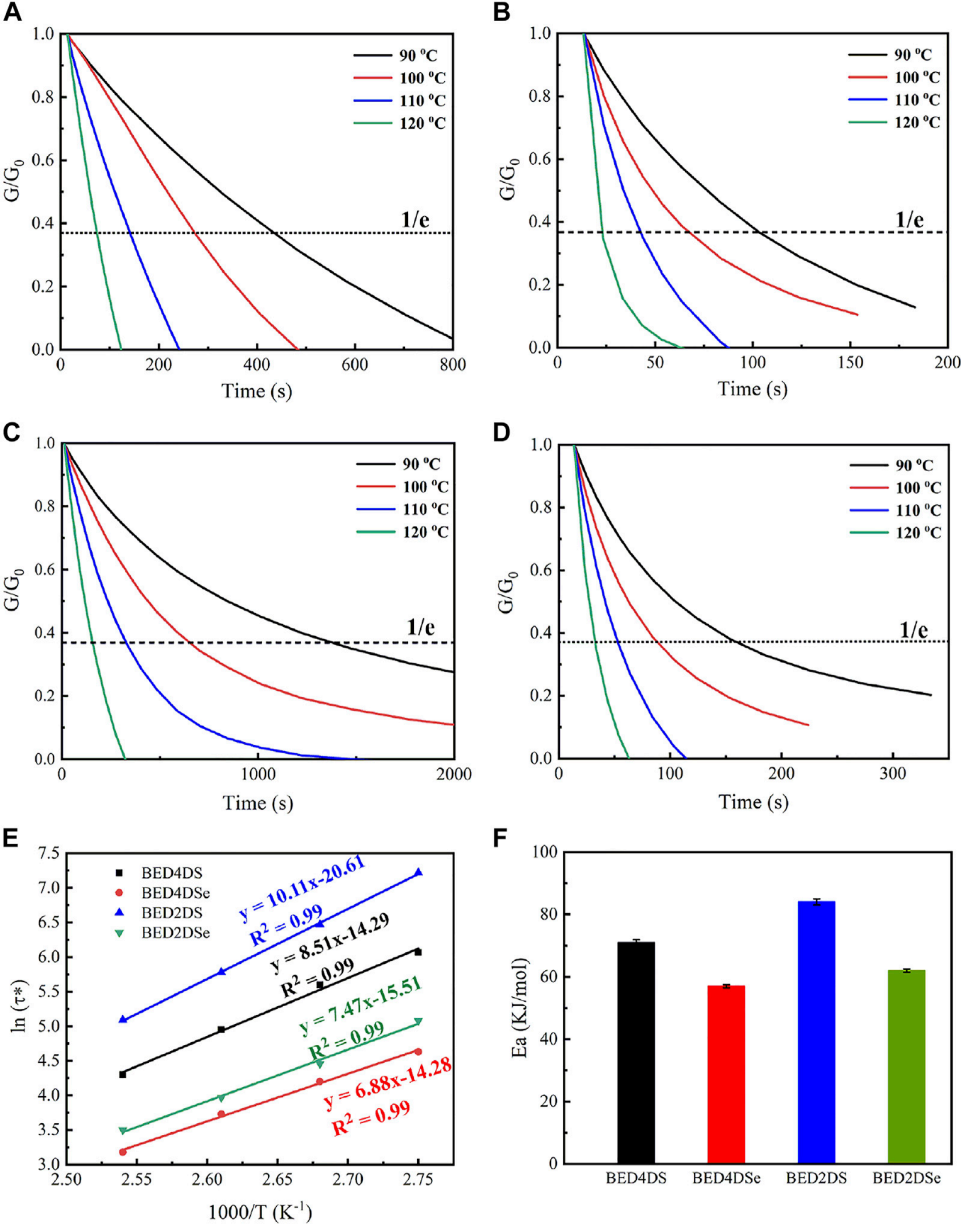
Normalized stress-relaxation curves at different temperatures for **(A)** BED4DS, **(B)** BED4DSe, **(C)** BED2DS, **(D)** BED2DSe; **(E)** linear fitting of the relaxation times by Arrhenius equation. **(F)** the activation energies of different samples.

The disulfide and diselenide bonds were dynamic covalent bonds that could undergo bond exchange reactions within the network at a certain temperature. As a result, the resin with a dynamic covalent bond was recyclable and repairable. DMA evaluated the samples stress-relaxation test. All of the samples, as shown in Figure 3, were able to relax stress and flow at temperatures above 90°C, and the normalized stress-relaxation curves at different temperatures (90°C, 100°C, 110°C, and 120 °C). The relaxation time was defined by the Maxwells model as the time it took for the modulus to relax to 1/e of its initial value, or G_(t)_/G_0_ = 0.37 (Tangthana-umrung et al., 2021; Lin et al., 2022; Sun et al., 2022). The relaxation times of BED4DSe and BED2DSe were 22 s and 34 s at 120°C, respectively, which were much shorter than the disulfide epoxy resin networks (BED4DS and BED2DS). The lower bond energies of the diselenide bond were attributed to the faster relaxation time (Kildahl 1995). At the same temperature, lower bond energies resulted in faster chain mobility of the network and faster exchange reaction speed. As shown in Figure 3E; Supplementary Figures S1–S4, the variation curve of relaxation time with temperature according to the Arrhenius equation, which is given by Eq. 2 (Chen et al., 2021; Spiesschaert et al., 2021; Zhao et al., 2021).

$$\text{τ}\left(\text{T}\right)\;={\text{ τ}}_{0}\text{exp}\left(\frac{{\text{E}}_{\text{a}}}{\text{RT}}\right)$$ (2)

where ${\text{τ}}_{0}\;$ is the relaxation time at infinite temperature, R is the universal gas constant, and *E*_a_ express the activation energy. The activation energy of four samples (BED4DS, BED4DSe, BED2DS, and BED2DSe) was calculated to be 71, 57, 84, and 62 kJ/mol, respectively, using the equation. When comparing the activation energies of disulfide and diselenide linkers in the epoxy network, it is clear that the activation energies of disulfide linkers were higher (up to 14 kJ/mol) implying that diselenide bonds are more dynamic. The activation energy of p-phenylamino networks (BED4DS and BED4DSe) was lower than that of o-phenylamino networks (BED2DS and BED2DSe), which could be attributed to the steric hindrance of o-phenylamino linkers. (Qian et al., 2019)

### Self-healing of dynamic epoxy resin networks

**FIGURE 4:**
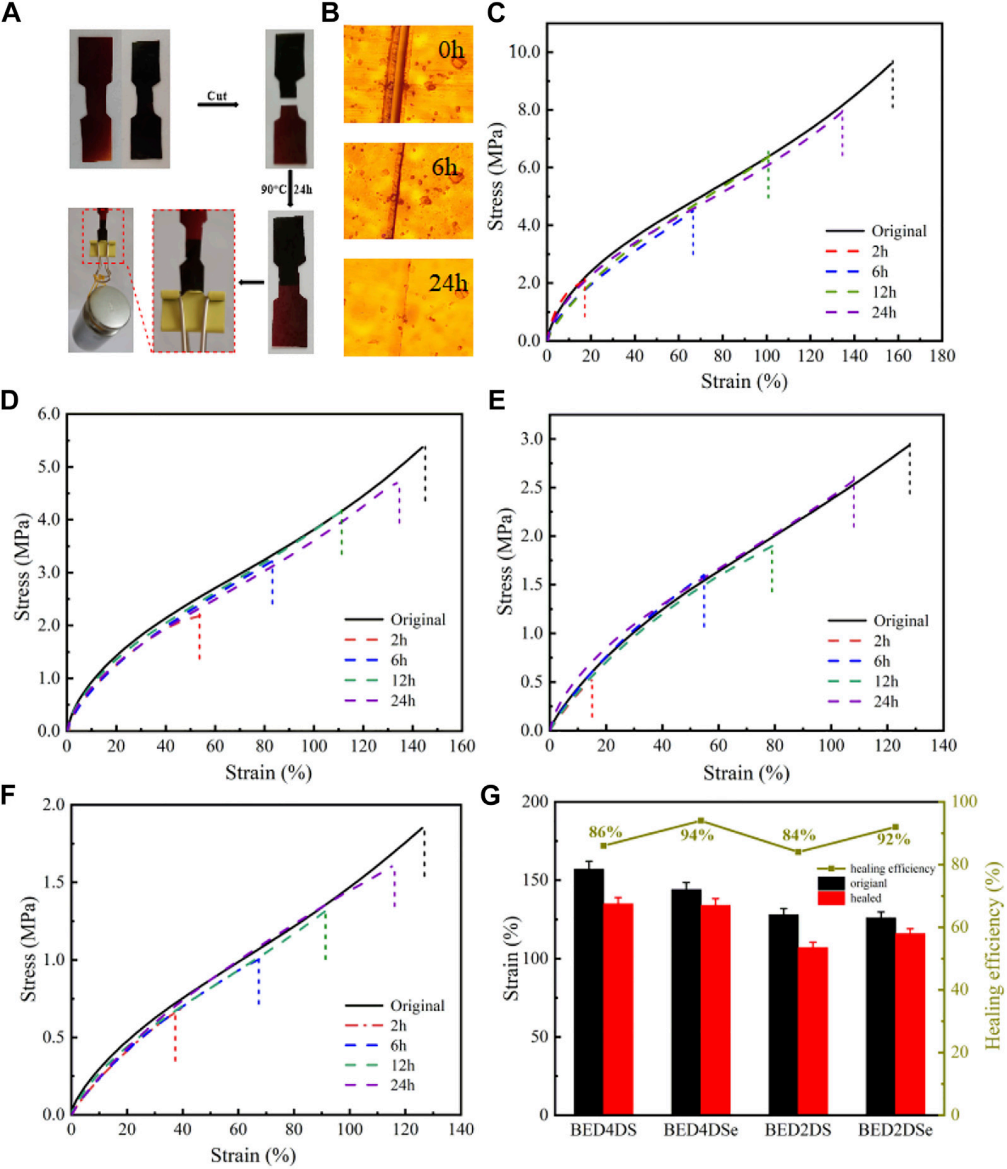
Self-healing of dynamic epoxy resin networks, **(A)** images of BED4DSe sample **(B)** optical microscopic images of the BED4DSe, **(C)** self-healing of the strain-stress curves for the BED4DS, **(D)** self-healing of the strain-stress curves for the BED4DSe; **(E)** self-healing of the strain-stress curves BED2DS, **(F)** self-healing of the strain-stress curves for the BED2DSe; **(G)** the self-healing properties of the four resins.

To verify the self-healing ability of the samples, two dumbbell samples (with different colors) were cut into two pieces using BED4DSe as an example. One of the dumbbell samples was stained with black dye, which is clearer to observe. The two halves were rejoined together using external forces (which ensured two halves of samples can be connected temporarily) and the sample was kept at 90°C for 24 h. As shown in Figure 4A, the healed sample was strong enough to withstand 200 g of weight without tearing. Supplementary Figures S6–S8 show that other samples also exhibit the same self-healing capability. The optical images of BED4DS were recorded during the process to allow for a more intuitive observation of the recovery result. A knife was used to cut the sample in half. The healed sample was then healed at 90°C. Figure 4B depicts the optical microscopic images after healing 0 h, 6 h, and 24 h of healing. At first, the significant scratch on the sample surface can be seen, whereas the cut healed clearly after healing at 90°C for 6 h. The trace completely disappeared within 24 h. This demonstrated that the epoxy resins with the dynamic covalent bond had self-healing properties when stimulated externally.

The sample was subjected to a stress-strain test to further study the self-healing performance. We cut the sample in half and rejoined it with our hands. The cracked sample was then placed in a 90°C oven for varying times (namely, 0 h, 2 h, 6 h, 12 h, and 24 h). The healing efficiency (η) was able to calculated by the following equation (Qi et al., 2021; Wei et al., 2021):

$$\eta \;=\;\frac{\varepsilon \;healed\;}{\varepsilon \;origianl\;}$$ (3)

Where the tensile strength of the healed and the original sample samples was represented by  ε _healed_ and ε _origianl_, respectively. The healing efficiency (η) is also shown in Figure 4E and the mechanical properties were listed in Supplementary Tables S1,S2. It could be summarized that by increasing the healing time, the mechanical properties of healed samples improved clearly. The healing efficiency of samples based on tensile strength was over 84% after healing at 90°C for 24 h. It is worth noting that the healing efficiency (η) of p-phenylamino networks (BED4DS and BED4DSe) has a higher efficiency than the o-phenylamino networks (BED2DS and BED2DSe), and the healing efficiency (η) of the diselenide linkers (BED4DSe and BED2DSe) higher efficiency than the disulfide linkers (BED4DS and BED2DS). The above phenomena occurred as a result of the atom’s structure and low activation energy (Lei et al., 2014). In the same condition, the lower activation energy, the faster relaxation speed, and the higher healing efficiency. In the periodic table, sulfur and selenium are in the same family and the atomic radius gradually increased. The structure of selenium atoms is less unstable and more prone to bond breakage under external stimulation for rapidly exchanging recombination reactions. Then, the η for 6 h, 12 h, and 24 h displayed the same law. The epoxy network clearly contained the diselenide bond better (Qian et al., 2019).

## Conclusion

In conclusion, four self-healing epoxy networks were created through the reaction of diselenide and disulfide dynamic linkers reaction with bisphenol A epoxy resins (128) and poly (ethylene glycol) diglycidyl ether (DER736). FTIR confirmed the chemical structure, and DSC, DMA, and TGA were used to characterize the thermal and mechanical properties. The results show that the glass transition temperature (*T*_g_) values, mechanical properties, crosslinking density (*v*_*e*_), and thermal stability of disulfide linkers networks were higher than those of diselenide linkers networks, and the p-phenylamino networks were higher than the o-phenylamino networks. The activation energies of disulfide linkers were higher than those of diselenide linkers (up to 14 kJ/mol) indicating that diselenide bonds may be more dynamic. Finally, the self-healing properties of these networks were investigated and the healing efficiency of diselenide linkers was found to be significantly higher than that of disulfide linkers networks. These advantageous properties of diselenide and disulfide dynamic linkers in epoxy networks system will provide a method for designing and preparing the right self-healing epoxy networks for the right application and processing technology.

## Data Availability

The original contributions presented in the study are included in the article/supplementary material, further inquiries can be directed to the corresponding authors.

## References

[B1] Azcune I. Odriozola I. (2016). Aromatic disulfide crosslinks in polymer systems: Self-healing, reprocessability, recyclability and more. Eur. Polym. J. 84, 147–160. 10.1016/j.eurpolymj.2016.09.023 10.1016/j.eurpolymj.2016.09.023 | Google Scholar

[B2] Billiet S. Hillewaere X. K. D. Teixeira R. F. A. Du Prez F. E. (2013). Chemistry of crosslinking processes for self-healing polymers. Macromol. Rapid Commun. 34 (4), 290–309. 10.1002/marc.201200689 PubMed Abstract | 10.1002/marc.201200689 | Google Scholar 23255325

[B3] Burattini S. Greenland B. W. Chappell D. Colquhoun H. M. Hayes W. (2010). Healable polymeric materials: A tutorial review. Chem. Soc. Rev. 39 (6), 1973–1985. 10.1039/b904502n PubMed Abstract | 10.1039/b904502n | Google Scholar 20502798

[B4] Chang H. Kim M. S. Huber G. W. Dumesic J. A. (2021). Design of closed-loop recycling production of a Diels-Alder polymer from a biomass-derived difuran as a functional additive for polyurethanes. Green Chem. 23 (23), 9479–9488. 10.1039/d1gc02865k PubMed Abstract | 10.1039/d1gc02865k | Google Scholar 35237099 PMC8884468

[B5] Chen F. Cheng Q. Gao F. Zhong J. Shen L. Lin C. (2021). The effect of latent plasticity on the shape recovery of a shape memory vitrimer. Eur. Polym. J. 147, 110304. 10.1016/j.eurpolymj.2021.110304 10.1016/j.eurpolymj.2021.110304 | Google Scholar

[B6] Chen F. Gao F. Zhong J. Shen L. Lin Y. (2020). Fusion of biobased vinylogous urethane vitrimers with distinct mechanical properties. Mat. Chem. Front. 4 (9), 2723–2730. 10.1039/d0qm00302f 10.1039/d0qm00302f | Google Scholar

[B7] Chung D. (2019). A review of multifunctional polymer-matrix structural composites. Compos. Part B Eng. 160, 644–660. 10.1016/j.compositesb.2018.12.117 10.1016/j.compositesb.2018.12.117 | Google Scholar

[B8] de Luzuriaga A. R. Martin R. Markaide N. Rekondo A. Cabañero G. Rodríguez J. (2016). Epoxy resin with exchangeable disulfide crosslinks to obtain reprocessable, repairable and recyclable fiber-reinforced thermoset composites. Mat. Horiz. 3 (3), 241–247. Google Scholar

[B9] Fan F. Ji S. Sun C. Liu C. Yu Y. Fu Y. (2018). Wavelength-controlled dynamic metathesis: A light-driven exchange reaction between disulfide and diselenide bonds. Angew. Chem. Int. Ed. 57 (50), 16426–16430. 10.1002/anie.201810297 10.1002/anie.201810297 | Google Scholar 30345597

[B10] García M. Jones O. Virwani K. McCloskey D. Boday J. ter Huurne M. (2014). Recyclable, strong thermosets and organogels via paraformaldehyde condensation with diamines. Science 344 (6185), 732–735. 10.1126/science.1251484 PubMed Abstract | 10.1126/science.1251484 | Google Scholar 24833389

[B11] Guo X. Gao F. Chen F. Zhong J. Shen L. Lin C. (2021). Dynamic enamine-one bond based vitrimer via amino-yne click reaction. ACS Macro Lett. 10 (10), 1186–1190. 10.1021/acsmacrolett.1c00550 PubMed Abstract | 10.1021/acsmacrolett.1c00550 | Google Scholar 35549045

[B12] He C. Shi S. Wang D. Helms B. A. Russell T. P. (2019a). Poly(oxime-ester) vitrimers with catalyst-free bond exchange. J. Am. Chem. Soc. 141 (35), 13753–13757. 10.1021/jacs.9b06668 PubMed Abstract | 10.1021/jacs.9b06668 | Google Scholar 31433176

[B13] He X. Zhong J. Cao Z. Wang J. Gao F. Xu D. (2019b). An exploration of the Knoevenagel condensation to create ambient curable coating materials based on acetoacetylated castor oil. Prog. Org. Coatings 129, 21–25. 10.1016/j.porgcoat.2018.12.015 PubMed Abstract | 10.1016/j.porgcoat.2018.12.015 | Google Scholar

[B14] Imato K. Nishihara M. Kanehara T. Amamoto Y. Takahara A. Otsuka H. (2012). Self-healing of chemical gels cross-linked by diarylbibenzofuranone-based trigger-free dynamic covalent bonds at room temperature. Angew. Chem. Int. Ed. 51 (5), 1138–1142. 10.1002/anie.201104069 PubMed Abstract | 10.1002/anie.201104069 | Google Scholar 22052848

[B15] Ji S. Cao W. Yu Y. Xu H. (2015). Visible-light-induced self-healing diselenide-containing polyurethane elastomer. Adv. Mat. 27 (47), 7740–7745. 10.1002/adma.201503661 10.1002/adma.201503661 | Google Scholar 26484966

[B16] Kar G. P. Saed M. O. Terentjev E. M. (2020). Scalable upcycling of thermoplastic polyolefins into vitrimers through transesterification. J. Mat. Chem. A 8 (45), 24137–24147. 10.1039/d0ta07339c 10.1039/d0ta07339c | Google Scholar

[B17] Kildahl N. K. (1995). Bond energy data summarized. J. Chem. Educ. 72 (5), 423. 10.1021/ed072p423 10.1021/ed072p423 | Google Scholar

[B18] Kim S.-M. Jeon H. Shin S.-H. Park S.-A. Jegal J. Hwang S. Y. (2018). Superior toughness and fast self-healing at room temperature engineered by transparent elastomers. Adv. Mat. 30 (1), 1705145. 10.1002/adma.201705145 PubMed Abstract | 10.1002/adma.201705145 | Google Scholar 29131415

[B19] Lafont U. van Zeijl H. van der Zwaag S. (2012). Influence of cross-linkers on the cohesive and adhesive self-healing ability of polysulfide-based thermosets. ACS Appl. Mat. Interfaces 4 (11), 6280–6288. 10.1021/am301879z PubMed Abstract | 10.1021/am301879z | Google Scholar 23082869

[B20] Le Goff N. Fomba I. Prost E. Merlier F. Haupt K. Duma L. (2020). Renewable plant oil-based molecularly imprinted polymers as biopesticide delivery systems. ACS Sustain. Chem. Eng. 8 (42), 15927–15935. 10.1021/acssuschemeng.0c05145 10.1021/acssuschemeng.0c05145 | Google Scholar

[B21] Lei Z. Q. Xiang H. P. Yuan Y. J. Rong M. Z. Zhang M. Q. (2014). Room-temperature self-healable and remoldable cross-linked polymer based on the dynamic exchange of disulfide bonds. Chem. Mat. 26 (6), 2038–2046. 10.1021/cm4040616 10.1021/cm4040616 | Google Scholar

[B22] Li M. Zhang H. Gao F. Tang Z. Zeng D. Pan Y. (2019). A cyclic cinnamate dimer mechanophore for multimodal stress responsive and mechanically adaptable polymeric materials. Polym. Chem. 10 (7), 905–910. 10.1039/c8py01654b 10.1039/c8py01654b | Google Scholar

[B23] Li T. Xie Z. Xu J. Weng Y. Guo B.-H. (2018). Design of a self-healing cross-linked polyurea with dynamic cross-links based on disulfide bonds and hydrogen bonding. Eur. Polym. J. 107, 249–257. 10.1016/j.eurpolymj.2018.08.005 10.1016/j.eurpolymj.2018.08.005 | Google Scholar

[B24] Lin Y. Chen Y. Yu Z. Huang Z. Lai J.-C. Tok J. B. H. (2022). Reprocessable and recyclable polymer network electrolytes via incorporation of dynamic covalent bonds. Chem. Mat. 34 (5), 2393–2399. 10.1021/acs.chemmater.1c04396 10.1021/acs.chemmater.1c04396 | Google Scholar

[B25] Ling L. Li J. Zhang G. Sun R. Wong C.-P. (2018). Self-healing and shape memory linear polyurethane based on disulfide linkages with excellent mechanical property. Macromol. Res. 26 (4), 365–373. 10.1007/s13233-018-6037-9 10.1007/s13233-018-6037-9 | Google Scholar

[B26] Liu F. Li F. Deng G. Chen Y. Zhang B. Zhang J. (2012). Rheological images of dynamic covalent polymer networks and mechanisms behind mechanical and self-healing properties. Macromolecules 45 (3), 1636–1645. 10.1021/ma202461e 10.1021/ma202461e | Google Scholar

[B27] Liu X. Song X. Chen B. Liu J. Feng Z. Zhang W. (2022). Self-healing and shape-memory epoxy thermosets based on dynamic diselenide bonds. React. Funct. Polym. 170, 105121. 10.1016/j.reactfunctpolym.2021.105121 10.1016/j.reactfunctpolym.2021.105121 | Google Scholar

[B28] Liu Y.-Y. He J. Li Y.-D. Zhao X.-L. Zeng J.-B. (2020). Biobased, reprocessable and weldable epoxy vitrimers from epoxidized soybean oil. Industrial Crops Prod. 153, 112576. 10.1016/j.indcrop.2020.112576 10.1016/j.indcrop.2020.112576 | Google Scholar

[B29] Luo X. Gao F. Chen F. Cheng Q. Zhao J. Wei X. (2020). Organic-inorganic hybrid coating materials derived from renewable soybean oil and amino silanes. RSC Adv. 10 (27), 15881–15887. 10.1039/d0ra01279c PubMed Abstract | 10.1039/d0ra01279c | Google Scholar 35493674 PMC9052391

[B30] Luo Y. Wang Y. Xia C. Ahmad A. Yang R. Li X. (2022). Eco-friendly soy protein isolate-based films strengthened by water-soluble glycerin epoxy resin. Prog. Org. Coatings 162, 106566. 10.1016/j.porgcoat.2021.106566 10.1016/j.porgcoat.2021.106566 | Google Scholar

[B31] Martín R. Rekondo A. Echeberria J. Cabañero G. Grande H. J. Odriozola I. (2012). Room temperature self-healing power of silicone elastomers having silver nanoparticles as crosslinkers. Chem. Commun. 48 (66), 8255–8257. Google Scholar 10.1039/c2cc32030d 22543710

[B32] Qi X. Zhang J. Zhang L. Yue D. (2021). Bio-based, robust, shape memory, self-healing and recyclable elastomers based on a semi-interpenetrating dynamic network. J. Mat. Chem. A 9 (45), 25399–25407. 10.1039/d1ta06299a 10.1039/d1ta06299a | Google Scholar

[B33] Qian Y. An X. Huang X. Pan X. Zhu J. Zhu X. (2019). Recyclable self-healing polyurethane cross-linked by alkyl diselenide with enhanced mechanical properties. Polymers 11 (5). 10.3390/polym11050773 PubMed Abstract | 10.3390/polym11050773 | Google Scholar PMC6572199 31052422

[B34] Rosu D. Mustata F. Tudorachi N. Musteata V. E. Rosu L. Varganici C. D. (2015). Novel bio-based flexible epoxy resin from diglycidyl ether of bisphenol A cured with castor oil maleate. RSC Adv. 5 (57), 45679–45687. 10.1039/c5ra05610a 10.1039/c5ra05610a | Google Scholar

[B35] Ruiz de Luzuriaga A. Solera G. Azcarate-Ascasua I. Boucher V. Grande H.-J. Rekondo A. (2022). Chemical control of the aromatic disulfide exchange kinetics for tailor-made epoxy vitrimers. Polymer 239, 124457. 10.1016/j.polymer.2021.124457 10.1016/j.polymer.2021.124457 | Google Scholar

[B36] Spiesschaert Y. Danneels J. Van Herck N. Guerre M. Acke G. Winne J. (2021). Polyaddition synthesis using alkyne esters for the design of vinylogous urethane vitrimers. Macromolecules 54 (17), 7931–7942. 10.1021/acs.macromol.1c01049 10.1021/acs.macromol.1c01049 | Google Scholar

[B37] Sun Y. Wang M. Wang Z. Mao Y. Jin L. Zhang K. (2022). Amine-cured glycidyl esters as dual dynamic epoxy vitrimers. Macromolecules 55 (2), 523–534. 10.1021/acs.macromol.1c01914 10.1021/acs.macromol.1c01914 | Google Scholar

[B38] Tangthana-umrung K. Poutrel Q. A. Gresil M. (2021). Epoxy homopolymerization as a tool to tune the thermo-mechanical properties and fracture toughness of vitrimers. Macromolecules 54 (18), 8393–8406. 10.1021/acs.macromol.1c00861 10.1021/acs.macromol.1c00861 | Google Scholar

[B39] Tretbar C. A. Neal J. A. Guan Z. (2019). Direct silyl ether metathesis for vitrimers with exceptional thermal stability. J. Am. Chem. Soc. 141 (42), 16595–16599. 10.1021/jacs.9b08876 PubMed Abstract | 10.1021/jacs.9b08876 | Google Scholar 31603321

[B40] Wang Z. Lu X. Sun S. Yu C. Xia H. (2019). Preparation, characterization and properties of intrinsic self-healing elastomers. J. Mat. Chem. B 7 (32), 4876–4926. 10.1039/c9tb00831d PubMed Abstract | 10.1039/c9tb00831d | Google Scholar 31411621

[B41] Wei X. Ge J. Gao F. Chen F. Zhang W. Zhong J. (2021). Bio-based self-healing coating material derived from renewable castor oil and multifunctional alamine. Eur. Polym. J. 160, 110804. 10.1016/j.eurpolymj.2021.110804 10.1016/j.eurpolymj.2021.110804 | Google Scholar

[B42] Zhang G. Zhou X. Liang K. Guo B. Li X. Wang Z. (2019). Mechanically robust and recyclable EPDM rubber composites by a green cross-linking strategy. ACS Sustain. Chem. Eng. 7 (13), 11712–11720. 10.1021/acssuschemeng.9b01875 10.1021/acssuschemeng.9b01875 | Google Scholar

[B43] Zhao S. Wang D. Russell T. P. (2021). Biobased dynamic polymer networks with rapid stress relaxation. ACS Sustain. Chem. Eng. 9 (33), 11091–11099. 10.1021/acssuschemeng.1c02826 10.1021/acssuschemeng.1c02826 | Google Scholar

[B44] Zhou F. Guo Z. Wang W. Lei X. Zhang B. Zhang H. (2018). Preparation of self-healing, recyclable epoxy resins and low-electrical resistance composites based on double-disulfide bond exchange. Compos. Sci. Technol. 167, 79–85. 10.1016/j.compscitech.2018.07.041 10.1016/j.compscitech.2018.07.041 | Google Scholar

[B45] Zhu Y. Gao F. Wei X. Cheng Q. Zhao J. Cao Z. (2020b). A novel bio-based coating material prepared from modified acetoacetylated castor oil and diisocyanate. Prog. Org. Coatings 138, 105397. 10.1016/j.porgcoat.2019.105397 10.1016/j.porgcoat.2019.105397 | Google Scholar

[B46] Zhu Y. Gao F. Zhong J. Shen L. Lin Y. (2020a). Renewable castor oil and DL-limonene derived fully bio-based vinylogous urethane vitrimers. Eur. Polym. J. 135, 109865. 10.1016/j.eurpolymj.2020.109865 10.1016/j.eurpolymj.2020.109865 | Google Scholar

[B47] Zuo H. Cao Z. Shu J. Xu D. Zhong J. Zhao J. (2019). Effect of structure on the properties of ambient-cured coating films prepared via a Michael addition reaction based on an acetoacetate-modified castor oil prepared by thiol-ene coupling. Prog. Org. Coatings 135, 27–33. 10.1016/j.porgcoat.2019.05.032 10.1016/j.porgcoat.2019.05.032 | Google Scholar

