# Supplementary material for: Exploring of the property of epoxy resins based on diselenide and disulfide dynamic linkers
Source: Front Chem. 2022 Sep 5;10:991010. doi: 10.3389/fchem.2022.991010 (PMC9484540; doi:10.3389/fchem.2022.991010)
Supplement: Supplementary file 1 [file DataSheet1.docx]

**Supporting Information For**

# Exploring of the property of epoxy resins based on diselenide and disulfide dynamic linkers

Xiao Wei, Feng Liu*, Xinru Guo, Fei Gao*, Yingjia Li, Dongtao Zhu, Zhi Zhou, Liang Shen*

Jiangxi Engineering Laboratory of Waterborne Coating, School of Chemistry and Chemical Engineering, Jiangxi Science &Technology Normal University, Nanchang 330013, Jiangxi, P. R. China

E-mail address: feng67807@163.com (F. Liu), feigao2016@jxstnu.com.cn (F. Gao); liangshen@jxstnu.com.cn (L. Shen).

**Table of Contents**

[1. Thermal stress relaxation of these polymer networks 2](#_Toc100008932)

[2.The healing efficiency of the samples 4](#_Toc100008933)

[3. ^1^H and ^13^CNMR Spectra 7](#_Toc100008934)

# Thermal stress relaxation of these polymer networks

**

**

**Figure S1** a) Stress relaxation curves of BED4DS at different temperatures and b) The relaxation times of BED4DS fitted to the Arrhenius equation.

**

**

**Figure S2** a) stress relaxation curves of BED4DSe at different temperatures and b) The relaxation times of BED4DSe fitted to the Arrhenius equation.

**

**

**Figure S3** a) stress relaxation curves of BED2DS at different temperatures and b) The relaxation times of BED2DS fitted to the Arrhenius equation.





**Figure S4** a) stress relaxation curves of BED2DSe at different temperatures and b) The relaxation times of BED2DSe fitted to the Arrhenius equation.

# 2.The healing efficiency of the samples

**
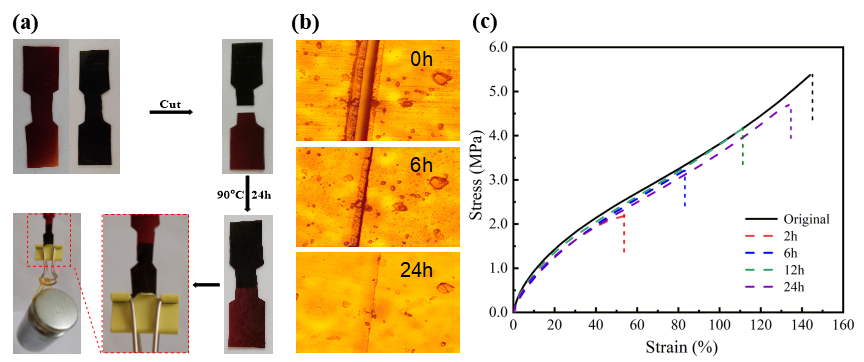
**

**Figure S5** a) The dumbbell sample of BED4DSe were cut into two halves and the healed one can subject a 200g weight; b) optical microscope image of BED4DSe of the self-healing process for different times; c) tensile properties of the healed samples at different times


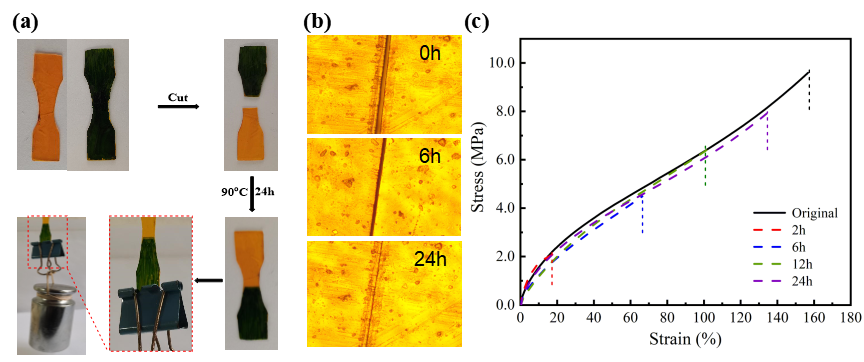


**Figure S6** a) The dumbbell sample of BED4DS were cut into two halves and the healed one can subject a 200g weight; b) optical microscope image of BED4DS of the self-healing process for different times; c) tensile properties of the healed samples at different times


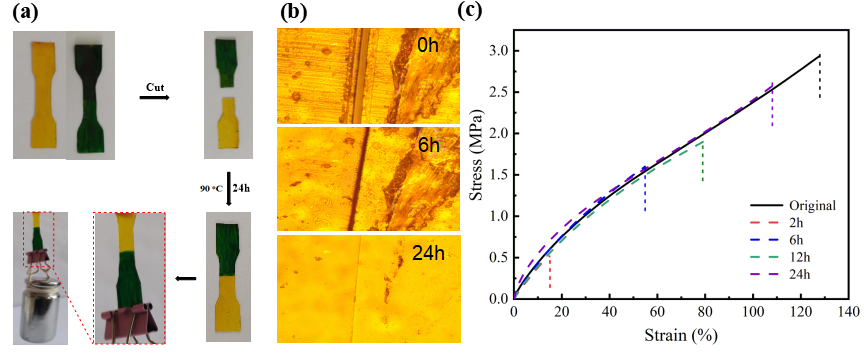


**Figure S7** a) The dumbbell sample of BED2DS were cut into two halves and the healed one can subject a 200g weight; b) optical microscope image of BED2DS of the self-healing process for different times; c) tensile properties of the healed samples at different times

**
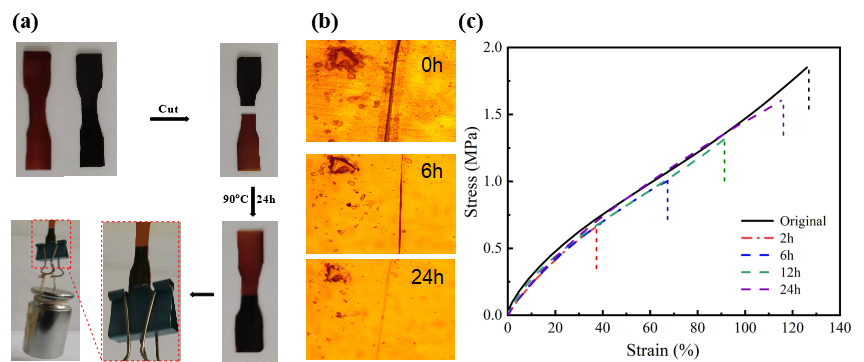
**

**Figure S8** a) The dumbbell sample of BED2DSe were cut into two halves and the healed one can subject a 200g weight; b) optical microscope image of BED2DSe of the self-healing process for different times; c) tensile properties of the healed samples at different times

**Table S1** the elongation at break of the original and the healed samples at different time

| Sample | $\varepsilon$_origianl_ (%) | $\varepsilon$_healed-2h_ (%) | $\varepsilon$_healed-6h_ (%) | $\varepsilon$_healed-12h_  (%) | $\varepsilon$_healed-24h_ (%) |
| --- | --- | --- | --- | --- | --- |
| BED4DS | 157 | 19 | 67 | 100 | 135 |
| BED4DSe | 144 | 56 | 86 | 114 | 134 |
| BED2DS | 128 | 15 | 54 | 79 | 107 |
| BED2DSe | 126 | 39 | 70 | 94 | 116 |

**Table S2** the healing efficiency (η) of the original at different healing time

| $\eta$  Sample | 0h  (%) | 2h  (%) | 6h  (%) | 12h  (%) | 24h  (%) |
| --- | --- | --- | --- | --- | --- |
| BED4DS | - | 12 | 43 | 64 | 86 |
| BED4DSe | - | 39 | 60 | 79 | 94 |
| BED2DS | - | 12 | 42 | 62 | 84 |
| BED2DSe | - | 31 | 55 | 75 | 92 |

# 3. ^1^H and ^13^CNMR Spectra

**

**

**Figure S9** ^1^H NMR spectrum of 4-DSe





**Figure S11** ^13^C NMR spectrum of 4-DSe

**

**

**Figure S10** ^1^H NMR spectrum of 2-DSe





**Figure S12** ^13^C NMR spectrum of 2-DSe
